# Progress in Neurology

**Published:** 1902-06-07

**Authors:** 


### PROGRESS IN NEUROLOGY.

(*Continued from page* 152.)

**Congenital Word-Blindness.**—In some further observations on this subject, Hinshelwood[12] said that he had come to the conclusion that there were memory centres in the brain for words, figures and letters; these centres are acquired, and are present on one side of the brain only, and consist of groups of specially developed cells. On no other theory could such cases as the following be explained, *e.g.*, where the memory for words only was blotted out, or where the scholar conversant with many languages became word-deaf to his mother tongue alone. In cases of word-blindness the only hope lay in re-education of the patient so that a corresponding area on the other side of the brain might develop and take up the work. In cases of congenital word-blindness the children could spell, count and repeat passages by heart, but had great difficulty in learning to read. In the attempt to read they would repeat aloud the letters of the word, thus making use of their auditory memory centre, which was quite intact. Such cases were probably due to defective development in the cells of the brain concerned in the usual memory for words and letters.

**Tabes Due to Congenital Syphilis.**—James[13] records three cases of early tabes due to inherited syphilis, occurring in one family. The eldest, aged 20 years, had Argyll-Robertson pupils, paralysis of the external rectus, absent knee-jerks, static ataxy, slight but definite anæsthesia over the lower extremities. The ocular paralysis had been present for a week, whilst the other tabetic symptoms had probably been coming on for about a year. The upper central incisors were widely separated, and narrowed below at their cutting edges. Otherwise there was no evidence of congenital syphilis, and there was no history of syphilitic manifestations during infancy or childhood. The next child, a boy aged 17 years, had a square head, prominent forehead, and saddle-shaped nose, scars at the angles of the mouth and pegged upper central incisors. There were gross central choroido-retinal changes in the right eye, with vitreous opacities. The right pupil was sluggish to direct light, but was active consensually. The knee-jerks were sluggish, there was no anæsthesia or incoördination. The next, a girl, aged 15 years, had a saddle-shaped nose and pegged upper central

DAMAGE

incisors. The pupils were unequal, very feebly reactive to light, but reacted well to accommodation. The knee-jerks were sluggish. There was no anæsthesia or incoördination. The fourth and fifth children presented evidences of congenital syphilis but no signs of tabes, whilst the sixth and seventh were healthy. Thus of seven children the eldest presented well-marked tabes, whilst the second and third had Argyll-Robertson pupils and sluggish knee-jerks. Such cases are very uncommon, and much rarer even than infantile general paralysis due to congenital syphilis. Whether Argyll-Robertson pupils and absent knee-jerks can remain during a long life the sole evidence of congenital syphilis is unknown.

**Prognosis of diseases of the nervous system** was discussed by Bury in his [14]Bradshaw lecture. In the case of disease produced by toxins, both selective action and immunity play an important part in prognosis. Thus the poison of syphilis shows a preference for the vascular structures at the base of the brain, for branches of the cerebral and spinal vessels, for the afferent conducting paths of the spinal cord, and for the cortical cells in the anterior part of the brain. The usual statement that organic affections of the nervous system run a more favourable course when due to syphilis than when started by other agencies must be taken with considerable qualifications. Thus if blocking of an artery take place, the brain tissue is permanently damaged, whether the clot be of syphilitic or other origin. Again, myelitis not due to syphilis may occasionally entirely pass away. Further, the prognosis in syphilitic cases in regard to the ultimate outcome may be worse than that of any other poison. Thus, recovery from a syphilitic meningo-myelitis may be followed by a breakdown in some other part of the nervous system. In this respect syphilis presents a contrast to the specific fevers; thus if disseminated myelitis follow measles and the patient recovers, we may be pretty confident that no subsequent nervous affection will develop as a result of the original infection. In syphilis, on the contrary, there appears to be an infinite capacity for future developments of its toxins. The mere size of a lesion appears to have but little influence on the progress of disease. Absence of visible pathological changes is no criterion of the possibility of cure. Thus the lesions underlying paralysis agitans, epilepsy, and spasmodic torticollis are unknown, and yet we cannot regard these diseases as curable. When the bulbar neurons presiding over the functions of respiration and deglutition are involved, life is seriously endangered. When other cells and fibres are implicated, the question of loss of function has to be considered rather than that of any immediate danger to life. Other things being equal, lesions of the peripheral nerves are more quickly and completely recovered from than those of the central nervous system; and lesions of the brain, at least as regards the degree to which function is impaired, are less serious than those of the cord. Restoration of function may be said to be due either to the recovery of nerve tissue which is only partially damaged, or to the taking up by adjacent or distant structures of the functions which are lost. The difficulties of prognosis are increased by the fact that symptoms vary in different persons quite apart from the severity, distribution, and nature of the lesion. For instance, with the same amount of irritation of sensory fibres the perception of pain in one person is extreme, in another very slight. Morbid anatomy does not always explain why paralysis is profound in one person, moderate or only slight in degree in another, or why the degree of muscular spasm varies so much in affections of the upper neuron. Symptoms, again, are due as much to normal physiological functional activity imperfectly acquired as to the actual loss of function occasioned by the lesion; they result not only from the changes at the seat of the lesion, but also from the normal or perverted action of healthy neurons as an indirect effect of the lesion. It is, then, the symptoms which testify to active phases of disease which, for purposes of prognosis, require to be carefully studied; and prognosis depends less on our knowledge of pathology than on the accuracy of our experience with regard to symptomatology. Thus the downward progress of chronic nervous diseases such as disseminated sclerosis, locomotor ataxy, general paralysis, and myelitis may be arrested at any stage, and while, as a rule, it is only temporary, it may be permanent. These variations in, *e.g.*, general paralysis support the view of a general toxic condition rather than that there is a primary and progressive decay of nerve elements.

[12] Lancet, Dec. 7, 1901. [13] Lancet, Dec. 28, 1901. [14] Brit. Med. Jour., Nov. 9, 1901.

